# Inhibitory Effect of Nano-Formulated Extract of *Passiflora incarnata* on Dalton’s Lymphoma Ascites-Bearing *Swiss albino* Mice

**DOI:** 10.3390/pharmaceutics17020270

**Published:** 2025-02-18

**Authors:** Balasubramanian Deepika, Gopalarethinam Janani, Devadass Jessy Mercy, Saranya Udayakumar, Agnishwar Girigoswami, Koyeli Girigoswami

**Affiliations:** 1 Faculty of Allied Health Sciences, Chettinad Hospital and Research Institute, Chettinad Academy of Research and Education, Chettinad Health City, Kelambakkam 603103, India; deepikabalu70@gmail.com (B.D.); jananigopalarethinam98@gmail.com (G.J.); jessy331mercy@gmail.com (D.J.M.); usaranyaudayakumar@gmail.com (S.U.); 2 Centre for Global Health Research, Saveetha Medical College, Saveetha Institute of Medical and Technical Sciences, Thandalam 602101, India

**Keywords:** *Passiflora incarnata*, ethanolic extract, nanoformulation, antitumor activity, Dalton’s lymphoma ascites

## Abstract

**Background/Objectives:** This study explored the antitumor effect of Passiflora incarnata leaves’ nanoformulation (N-EEP) in fibroblasts, A375 cell lines, and in vivo using Dalton’s lymphoma ascites (DLA)-bearing mice. **Methods:** N-EEP treatment could significantly slow scratch closing in A375 cells compared to in the extract itself (EEP). **Results:** The hemolytic assay showed that N-EEP had less than 2% hemolysis, making the formulation highly biocompatible. In vivo N-EEP administration delayed the tumor growth rate, reduced weight gain, and increased the tumor-bearing mice’s life span. Furthermore, the ascitic cells were aspirated from the tumor and investigated for various gene expressions. The tumor suppressor gene p53, which plays a significant role in the mitochondrial-mediated apoptosis pathway, was found to be elevated in animals treated with N-EEP. We assessed the cytotoxicity of isolated DLA cells from induced mice using both the trypan blue and MTT assays, while apoptotic studies were conducted using Hoechst staining. Results from the trypan blue and MTT assays indicated that nearly 80% of the cells were killed by N-EEP treatment (200 μg/mL). Additionally, apoptosis, characterized by condensed nuclei, was observed after N-EEP treatment, confirming that one of the modes of cell death was caspase-dependent apoptosis. **Conclusions:** Our study suggests that N-EEP delayed the growth of DLA by upregulating p53 gene expression and inducing apoptosis.

## 1. Introduction

Natural products have shown their profound application in the remedy of various diseases like cancer [1], amyloidosis [2,3], wound healing [4], cardiovascular disease [5], pulmonary diseases [6], etc. Along with identifying novel chemical entities for therapeutic use, natural products serve as a crucial basis for discovering potential lead compounds, which can be further developed into more effective drugs through structural modifications. Despite their diverse and intricate chemical structures, plant secondary metabolites are often regarded as having superior biological activity and drug-like properties compared to those from purely synthetic sources. As a result, compounds of natural origin are considered more promising candidates for advancing drug development. Vinca alkaloids, epipodophyllotoxins, taxanes, and camptothecin are known phytochemicals that can cause apoptosis in cancer cells [7]. Nanoencapsulation of the natural compounds is also becoming popular for its demonstration of superior anticancer activities [8].

Incurable diseases like cancer predominantly cause worldwide death; according to global cancer statistics, 3 million people are expected to die by 2030 due to cancer [9]. Researchers conducted a five-year study on early-age cancer incidence, finding that age-adjusted overall cancer incidence and mortality rates remained stable throughout the study. However, the age-adjusted incidence rates showed a significant decline for ependymoma, melanoma, lung, bronchus, and tracheal carcinomas, unspecified malignant neoplasms, and non-Hodgkin’s lymphoma. In contrast, notable increases were observed in gastrointestinal tract cancers and non-Kaposi sarcomas [10]. On the other hand, an estimated 4,824,700 new cancer cases and 2,574,200 cancer-related deaths occurred in China in 2022 [11]. Thus, it is warranted that new therapeutic approaches involving natural products must come into the market for efficient management of this disease.

Plant-based extracts and formulations were tested as radiotherapeutic and chemotherapeutic agents against different types of cancer [12]. One of the promising methods for cancer treatment is chemotherapy. The role of naturally occurring pharmaceutical substances and bioactive components as chemotherapeutic agents has gained interest recently due to the occurrence of various drug-resistant cancer types [13]. The naturally occurring bioactive components reduce the toxicity and act on the tumor, thereby suppressing the side effects caused by chemotherapeutic agents [14]. The major drawback of using plant-based medicine is its lower bioavailability because most bioactive components are insoluble or sparingly soluble in water. Their larger molecular size also makes them less bioavailable, and nanoformulations have been used to overcome this limitation and enhance the targeted drug delivery [15,16]. Nanotechnology has captured the area of research in biomedicine due to its fascinating applications [17,18]. A spectrum of drug delivery systems has been used, such as liposomes, exosomes, microspheres, solid lipid nanoparticles, nanoemulsions, etc. [19,20,21], among which liposomes have gained much attention.

*Passiflora incarnata* is a perennial vine that commonly grows in tropical places across the world and is also known as maypop, passion fruit, and wild apricot [22]. This plant possessed various biomedical activities, such as antioxidant, antimicrobial, and anti-depressant activity [23,24,25,26,27]. The phytochemical isolated from *Passiflora incarnata* exhibited anticancer activity in breast cancer cells and cell lines of multidrug-resistant osteosarcoma [28,29]. In our previous study, we identified that the nanoformulation of ethanolic extract from *Passiflora incarnata* (N-EEP) has anticancer activity against lung cancer, oral cancer, and skin cancer cell lines, and we have also found that the toxicity of the ethanolic extract (EEP) is reduced by the N-EEP in vitro as well as in vivo model [30]. Our present study aimed to identify the anticancer potential of EEP and N-EEP in Dalton’s lymphoma ascites (DLA)-bearing mice. We have identified the major components of the EEP using GC-MS and observed the surface morphology of EEP and N-EEP using scanning electron microscopy (SEM). The in vitro study was conducted using a Chinese hamster lung fibroblast (V79) cell line and a human melanoma cell line (A375) to observe the cell migration assay in treated and untreated groups. DLA-tumor-induced mice were taken, and cells were aspirated from the liquid tumor to conduct in vitro studies, such as trypan blue assay, MTT assay, and Hoechst staining. Biocompatibility was checked using a hemolysis assay and human blood. Finally, the Swiss albino mice were injected with DLA cells to induce the tumor, and the regression was observed after alternate-day injection with EEP and N-EEP.

## 2. Materials and Method

### 2.1. Materials

*Passiflora incarnata* was procured from a local vendor and maintained in the herbal garden of our institute (Chettinad Academy of Research and Education). A registered taxonomist did the plant identification and authentication. The source of DLA cells was Amala Cancer Research Centre, Thrissur, Kerala, India. A375 and V79 cells were ordered from the National Centre for Cell Science, Pune. Antibiotics and fetal bovine serum were purchased from Gibco, USA. MTT (3-(4,5-dimethylthiazol-2-yl)-2,5-diphenyltetrazolium bromide dye), DMEM (Dulbecco’s Modified Eagle Medium), PBS (phosphate buffer saline) tablets, trypan blue, cholesterol, isopropanol, acetone, methanol, Hi-cDNA synthesizing kit (MBT076-25R), and ethidium bromide (EtBr) were purchased from HiMedia, India. Diethyl pyrocarbonate (DEPC) was purchased from SRL, India. Other reagents such as Hoechst 33342, Trizol, and dimethyl sulfoxide were purchased from SRL, India. The primers for gene expression studies were purchased from Sigma Aldrich, India and the DreamTaq Green PCR Master Mix (2X) from ThermoFisher Scientific, India.

### 2.2. Preparation and Characterization of EEP and N-EEP

The preparation of the ethanolic extract of *Passiflora incarnata* leaves, nanoformulation, and the characterization of the ethanolic extract was conducted according to Deepika et al. 2024 [30]. The GC-MS analysis of EEP for identifying secondary metabolites was performed in an Agilent Technologies 7890B gas chromatographer coupled with a 5977B mass spectrophotometer. The retention time was 42 min in a DB-5ms column, 30 m × 0.25 μm. NIST-based automated spectral deconvolution and identification software was utilized to identify the active constituents of the sample. For SEM analysis, the samples were drop-cast on grease-free coverslips (glass) and dried thoroughly in a dirt-free atmosphere. The samples were analyzed at 10 kV using a Quanta 200 F SEM.

### 2.3. Scratch Assay

A scratch assay was executed according to Shurfa et al. 2023 [31] with slight modification. Approximately 10^6^ A375 and V79 cells were cultured in a 35 mm tissue culture plate containing complete DMEM (DMEM, 10% serum, and 1% antibiotic antimycotic solution) and incubated with 5% CO_2_ at 37 °C for 24 h. After incubation, a small scratch was made with a 100 μL pipette tip at the bottom of the plate, and images were taken with the help of the inverted microscope. Treatment was carried out with the same EEP and N-EEP (100 μg/mL) concentrations, and an untreated control plate was maintained simultaneously. Every 24 h, an image was captured at the region where the scratch was made in all the treated and control plates until the scratch closed entirely in the control plate. The Magnus software (MagnusAnalytics MagVision, version: x64, 4.11.17852.20201017) was used to estimate the area closed on the scratch. The percentage of unclosed areas was calculated using the formula given below [31].

$$Percentage\;of\;area\;unclosed=\frac{Af-Ai}{Ai}\times 100$$

Af is the final unclosed area

Ai is the initial unclosed area

Percentage of closed area = 100 − the percentage of unclosed area

### 2.4. Biocompatibility Assay

With slight modification, a hemolysis assay was conducted following Girigoswami et al. 2024 [32] to check the extracts’ biocompatibility (EEP and N-EEP). The experiment was carried out after obtaining appropriate ethical clearance from the Institutional Human Ethical Committee (IHEC) (Ref No: IHEC-II/0402/23) on 13 September 2023. After obtaining informed consent from a healthy human volunteer, the rights to privacy of the volunteer (male, age 21 years old) were strictly maintained; 3 mL of blood was drawn from the vein and transferred to an EDTA-coated tube. To obtain the RBC pellet, centrifugation of the blood was conducted at 1500 rpm for 15 min. Further to the pellet, 2 mL of normal saline solution (0.9% NaCl) was added and centrifuged at 1500 rpm for 5 min to wash the lysed RBCs. Then, a dilution of 10 times was performed for the RBC with normal saline. Ten different tubes were prepared, and each tube contained 100 μL of RBC and 900 μL of normal saline diluted with different concentrations (25, 50, 100, 150, and 200 μg/mL) of EEP and N-EEP. Negative control (0.9% NaCl) and positive control (distilled water) tubes were also maintained simultaneously. Tube incubation was performed for 2 h at 37 °C, followed by further centrifugation at 12,000 rpm for 1 min. The supernatant OD was recorded at 541 nm, and the below-mentioned formula was used to calculate the percentage of hemolysis [32].

$$\%\;of\;haemolysis=\frac{\left(OD\;of\;the\;Sample\;treated-OD\;of\;the\;negative\;control\right)}{\left(OD\;of\;the\;positive\;control-OD\;of\;the\;negative\;control\right)}\times 100$$

### 2.5. Animals

The 10–12 week-old female Swiss albino mice, with an average weight of between 25 and 30 g, were retained in a standard polypropylene cage (55 cm × 32.7 cm× 19 cm). The animals were provided with pellet feed, and their bedding material was paddy husk. They were maintained in a 12 h light and dark cycle, with a surrounding temperature of 21–25 °C. The animals were housed in the Chettinad Hospital and Research Institution animal house. The experiment was conducted after procuring clearance from the Institutional Animal Ethical Committee (IAEC) (IAEC 2/Proposal: 121/A.Lr: 93/Dt: 16 September 2023) on 16 September 2023. The guidelines of the Committee for Control and Supervision of Experiments on Animals (CCSEA), India, were followed while conducting the animal experiment. The animals for the experimental groups were separated randomly a week before starting the experiment.

### 2.6. Induction of DLA in Mice

DLA cells were purchased from Amala Cancer Research Centre, Thrissur, Kerala, India, and transported inside the peritoneal cavity of two mice. DLA cells were withdrawn from the mice’s peritoneal cavity using a 26-gauge needle and diluted with PBS, pH = 7.4, to obtain 10^6^ cells. These cells were transferred into the peritoneum of the healthy mice. They were further propagated in the peritoneum of three more mice. These mice were kept undisturbed for 7–9 days for cancer development. Ascitic fluid was freshly withdrawn from these mice for the induction of cancer in experimental groups and also for in vitro studies [33].

### 2.7. Cytotoxicity Study

#### 2.7.1. Short-Term Toxicity Effect

Trypan blue is a dye that tints the dead cells as dark blue/purple and does not stain the live cells. In this assay, we used trypan blue to identify the short-term effect of EEP and N-EEP on DLA cells. DLA cells were collected from the peritoneal cavity of the DLA-induced mice 8 days after tumor induction. The cells were checked for a viability percentage of 98% before starting the experiment. A total of 10^6^ cells were added to tubes containing various concentrations of EEP and N-EEP diluted in 1 mL of PBS (pH 7.4) along with the control, which contained only saline. Then, the tubes were further incubated for 3 h at 37 °C; after incubation, 10 μL of the sample with the cells was transferred to a fresh container that had 10 μL of trypan blue (0.4%) and further incubated for 3 min. The total number of cells and the number of dead cells were counted with the help of a hemocytometer. The percentage of short-term toxicity (% of cytotoxicity) was calculated according to the formula given below [34].

$$\%\;of\;cytotoxicity=(\frac{No.of\;dead\;cells}{Total\;no.of\;cells})\times 100$$

#### 2.7.2. Long-Term Toxicity Effect

Cellular cytotoxicity assessment of EEP and N-EEP was monitored using the MTT assay according to Deepika et al. 2024 [30] with a few modifications. DLA cells were withdrawn using a syringe (aspirated) from the peritoneal cavity of the mice and washed further with sterile PBS. Then 10^6^ cells were seeded in each well of a 24-well plate containing 1 mL of DMEM provided with 10% FBS and 5% antibiotic. Further, the cells were treated with different concentrations of EEP and N-EEP and incubated for 24 h at 37 °C with 5% CO_2_ in a humidified atmosphere. After incubation, 100 μL of MTT (5 mg/mL) was added to the wells and further incubated for 4 h to develop formazan crystals. The formazan crystals were then dissolved entirely with dimethyl sulfoxide (DMSO), and the absorbance of the solution was noted at 570 nm employing a UV–vis spectrophotometer (Shimadzu UV-1800, Tokyo, Japan). The experiment was carried out in triplicate [30].

### 2.8. Hoechst Staining Methodology

Hoechst staining was performed in the DLA cells to identify the apoptotic cell death according to our previous study [35] with slight modification. DLA cells were withdrawn from the peritoneum of mice, washed with PBS suspended in DMEM medium in a 24-well plate, treated with 300 μg/mL of EEP, 150 μg/mL, and 300 μg/mL of N-EEP, and incubated for 24 h at 37 °C. Further, we collected the cells, washed them gently with PBS, fixed them in an acetone and methanol solution at a ratio of 1:1, and incubated them at 4 °C for 1 h. After incubation, the cells were transferred to a grease-free slide, and a cell smear was made. Heat fixing was carried out for the cell smear, and it was stained with 1 mM of Hoechst. The surplus stain was then removed with sterile PBS, and the slides were visualized with a 40X objective lens under an Olympus BX51 fluorescent microscope [35].

### 2.9. Tumor Induction and Treatment Protocol

#### 2.9.1. Tumor Induction

DLA cells (10^6^ cells/mice) were inoculated into the peritoneal cavity of the Swiss albino mice divided into four experimental groups, as mentioned in Table 1 (6 animals in each group). A total of 10^6^ DLA cells were injected into each animal, and the treatment started on day 3 via intraperitoneal injection. Group 1 was assigned as a control that received only saline, animals in Group 2 were treated with EEP, and Groups 3 and 4 were treated with N-EEP at low and high doses, respectively. The treatment was conducted thrice a week until the animal survived. Regularly, the animals were observed for their body weight, food and water intake, and tumor reduction. After 19 days of treatment, DLA cells were collected with a syringe from the peritoneal cavity of mice and further used for gene expression analysis [36,37,38].

#### 2.9.2. Gain in Body Weight

The average gain in the body weight of the mice after the induction of cancer was monitored every day; the average body weight gain was calculated.

#### 2.9.3. Tumor Size Analysis

The tumor’s size near the mice’s hind limb region was measured before and after tumor induction every alternate day until the animal survived for all the groups. The size was measured at two different places (r1 and r2) in the hind limb region of the mice. The formula used for the calculation of tumor volume is given below [36].

Volume of tumor = 4/3 π (r1)^2^ r2 cm^3^

#### 2.9.4. Lifespan Extension (Percentage)

The lifespan increase (ILS) in percentage was calculated using the following formula [38,39]:

$$\%ILS=\left(\frac{Lifespan\;of\;treated\;group}{Lifespan\;of\;control\;group}\right)-1\times 100$$

#### 2.9.5. Gene Expression Analysis

##### RNA Isolation

The total RNA was isolated using a phenol–guanidinium–thiocyanate-based Trizol reagent assay according to Ghosh et al. 2008 [40] with minor modification. Every piece of plasticware used for RNA isolation was soaked for 12 h in DEPC-treated water, dried, and autoclaved for further use. Dedicated pipettes were used for mRNA isolation, and the reagents were added in a biosafety cabinet (BSL level II) under sterile conditions to avoid any contamination from RNase, etc. After 19 days of treatment, 10^7^ cells were collected from the mice, and the cells were washed with sterile PBS three times. A total of 1 mL of the Trizol reagent was mixed with the cells and incubated for 5 min on ice; after incubation, 200 μL of chloroform was poured into the solution and mixed gently. Ice incubation for 3 min was carried out with the solution, followed by centrifugation at 12,000 rpm for 15 min at 4 °C. The aqueous phase is the upper transparent phase that was collected carefully without disturbing the other layers and transferred to the fresh tube. A total of 250 μL of ice-cold ethanol was transferred slowly and gently blended to the aqueous layer and collected, followed by ice incubation for 10 min. RNA pellet was obtained after centrifugation (10,000 rpm for 10 min, 4 °C) and the gentle discarding of the supernatant. The obtained RNA pellet was rinsed with 75% ethanol by adding 500 μL of ethanol and centrifuging at 10,000 rpm for 5 min at 4 °C. Complete air drying of the pellet was performed in a sterile environment and suspended in 20 μL of RNase-free water [40].

##### cDNA Synthesis

Immediately after RNA isolation, cDNA was prepared with the help of a Hi-cDNA synthesizing kit (MBT076-25R). The reagents were added in an RNase-free PCR tube following the manufacturer’s protocol in a sterile environment. Briefly, in a tube, we added oligo(dT) (1 μL), random hexamer (1 μL), random hexamer/oligo (dT) mix (2 μL), RNA isolated from cells after N-EEP, EEP treatment, the control mice (2 μL), and, finally, molecular biology grade water (4 μL). Then, the tube incubation was performed at 65 °C for 5 min, followed by immediate collection. Then the following reactants were added to the tubes: RT buffer (M-MulV) (4 μL), 10X solution for M-MuLV reverse transcriptase (RNase H) (2 μL), ribonuclease inhibitor (0.5 μL), and 10 mM of dNTP mix (2 μL). The volume makeup was carried out using molecular biology grade water to 20 μL for each tube. Tubes were then vortexed and kept for reverse transcription at 42 °C for 60 min, followed by 70 °C for 5 min for one cycle. The tubes were kept at 4 °C and stored at −20 °C [41].

##### Polymer Chain Reaction for Identification of Genes Expressed

We used the cDNA synthesized to study the expression of caspase 3, Bax, and p53. The housekeeping gene was GAPDH, and the primer sequence used to amplify the genes is mentioned in Table 2. The reaction mixture for PCR consisted of 0.5 μL of forward and reverse primer, 2 μL of template cDNA, 12.5 μL of PCR master mix (DreamTaq Green PCR Master Mix (2X)), and the total reaction volume of 25 μL was made with molecular biology grade water. The initial denaturation was at 95 °C for 5 min; 49 cycles were run for each gene, and the cycling condition for each gene is mentioned in Table 2. After 49 cycles, the final extension was kept at 72 °C for 5 min. The PCR product was kept at 4 °C and stored at −20 °C [33].

##### Electrophoresis for Visualization of the PCR Product

The agarose gel (1.5%) for electrophoresis was prepared using tris boric acid EDTA buffer, to which EtBr (3 μL) was added. Further, the gel was transferred to a completely sealed casting boat and kept undisturbed until the gel solidified. A total of 6 μL of the PCR product and sample loading dye (2 μL) were mixed thoroughly and loaded in the corresponding wells. The gel run was performed at 100 V for 30 min and observed under a UV transilluminator. The gel pictures were captured with the help of the GeNei capture imaging software presented by the Gel-Doc. The Image J software was used to calculate the intensity of the bands [41].

### 2.10. Statistical Analysis

The data were represented as mean ± standard deviation. The significance level was determined using one-way ANOVA between the groups, followed by post hoc tests (e.g., Tukey’s HSD) to identify the specific differences between the groups. Significance was considered statistically for a *p*-value of less than or equal to 0.05.

## 3. Results and Discussions

Plant-based therapeutic strategies have gained tremendous popularity nowadays, providing alternative treatment options for many communicable and non-communicable diseases. A combination of nanotechnology and natural product medicine has bloomed in the pharmaceutical market recently, opening avenues for research in this area [42]. In the present study, we have extracted the phytochemicals present in dried leaves of the *Passiflora incarnata* plant and liposome-encapsulated them to monitor the cancerous tumor regression in DLA-induced mice.

### 3.1. Characterization of EEP and N-EEP

The major bioactive components revealed by GC-MS analysis present in EEP were found to be 1-dodecene, benzene, 1,3-bis (1,1-dimethylethyl)-, 1-tetradecene, 2,4-di-tert-butylphenol, cetene, diethyl phthalate, E-15-heptadecenal, dodecane, 4,6-dimethyl, neophytadiene, 2-hexadecen-1-ol, 3,7,11,15-tetramethyl-, acetate, 7,9-di-tert-butyl-1-oxaspiro(4,5)deca-6,9-diene-2,8- dione, 6-octadecenoic acid, methyl ester, (Z)-, phytol, (Z)-docos-9-enenitrile, ethyl 13-docosenoate(ethyl erucate), 13-docosenamide, (Z)-, squalene and dodecyl acrylate, and stigmasterol [43]. The surface morphology of EEP was cylindrical, whereas the N-EEP has a shape like a truncated sphere (Figure S1B). The approximate size of EEP was 235 nm, and N-EEP was 122 nm. The N-EEP stability was tested at three different temperatures over 24 days, and we found that the N-EEP was stable for up to 24 days when stored at 4 °C (Figure S1C).

### 3.2. Cell Migration Analysis

**Figure 1:**
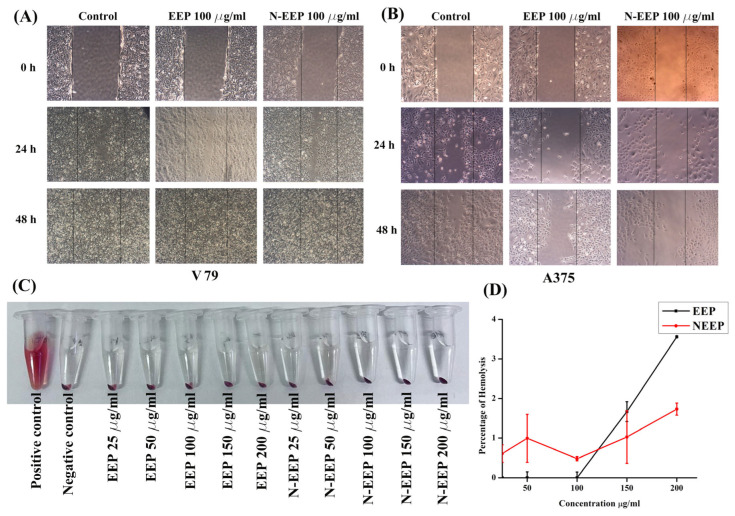
(**A**) Cell migration assay of V79 cell line; (**B**) A375 cell lines with 100 μg/mL of EEP and N-EEP treatment; magnification 20X (**C**) biocompatibility assay assessed by hemolysis conducted after 3 h incubation with various concentrations of EEP and N-EEP along with positive and negative control; and (**D**) percentage of hemolysis induced by EEP and N-EEP.

A scratch assay was used to monitor the migration of cells [31]. The cell migration analysis was conducted for EEP and N-EEP with A375 and V79 cells. Post-EEP treatment in the A375 cells, the percentage of area closure was 44.6 ± 8.58%, whereas for N-EEP it was 13.5 ± 5.9%. The cell migration was lowered in the N-EEP treated group significantly (*p* < 0.01) compared to the control and EEP treatment in A375 cells (Figure 1B). On the other hand, in V79, cell migration was inhibited in both the N-EEP and EEP-treated groups (Figure 1A). The percentage of area closed, as calculated by the Magnus software, is mentioned in Table 3 for normal fibroblasts (V79) and skin cancer (A375) cells.

Cell migration shows that the cells have communication between them, which inspires them to make colonies and grow further. Using the scratch assay, we can understand whether the cell–cell communication is retained or lost because this plays a major role in colony formation and tumor progression [44]. Our results have shown that N-EEP treatment could not only kill the cells, as visible by low cell density on day 3, but it also kept the scratch wide open. This indicated that cell-cell communication was lost or inhibited by N-EEP treatment in cancer cells. On the other hand, in normal fibroblast cells, N-EEP did not interfere with cell-cell communication, and the scratch was observed to be filled at a rate similar to that of untreated control. Thus, N-EEP can precisely target the cancer cells and encourage the inhibition of cell-cell signaling responsible for tumor growth. We obtained a similar result in our previous study, where the cancer cells had delayed scratch closure after treatment with doxorubicin-coated cerium oxide nanoparticles [31].

### 3.3. Biocompatibility Analysis

Regarding applying nanoformulation to remedy any disease, we should ensure the formulation is safe and biocompatible. Testing the drug in human blood enables us to monitor the biocompatibility [45]. After 3 h of incubation of RBC along with the EEP and N-EEP at various concentrations, the hemolysis percentage was calculated. The percentages of hemolysis at high concentrations (200 µg/mL) of EEP and N-EEP were 3.56 ± 0.003% and 1.83 ± 0.15%, respectively, which indicated that N-EEP was highly biocompatible in comparison with EEP (Figure 1C,D). Harini et al. formulated bupropion in a niosomal vesicle, checked the biocompatibility using a hemolysis assay, and found the formulation safe for human use [32].

### 3.4. Cytotoxicity Analysis

**Figure 2:**
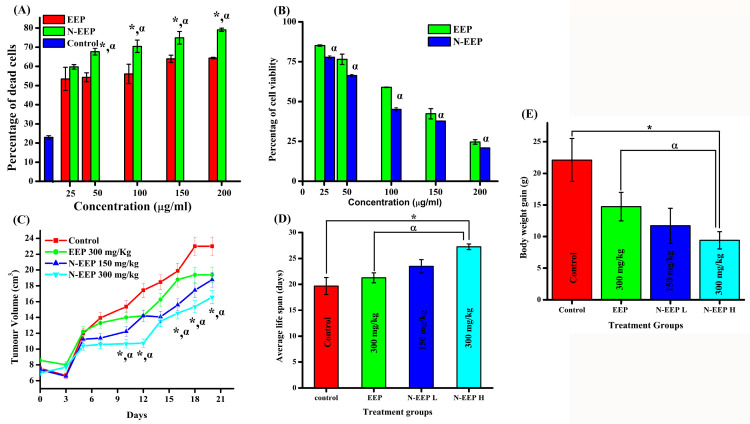
(**A**) Percentage of cell death after short-term toxicity analysis on DLA cells using trypan blue staining; (**B**) cell viability assessment using MTT analysis on DLA cells; (**C**) effect of EEP and N-EEP on tumor growth monitored by the increase in tumor volume with time; (**D**) average life span; and (**E**) body weight gain of the animals induced with DLA tumor and DLA tumor treated with EEP and N-EEP. (*—*p* < 0.01, in comparison with untreated control; α—*p* < 0.01, in comparison with the EEP treatment group at the same dose).

Trypan blue and MTT assays are commonly used to find cell viability [46,47]. Although trypan blue can only conclude whether the cell is alive or dead, an MTT assay can identify the living, metabolically active cells. Thus, if trypan blue is a dry test, the MTT assay is like a wet test in chemistry. We can use the rapid trypan blue test for a short-term, immediate effect of any drug on the cells. Whereas for a long-term effect, where at least one generation of cells must be multiplied, we can execute an MTT assay with 24 or 48 h of exposure to the drug. Cell death percentage of DLA cells after 3 h of treatment with various concentrations of EEP and N-EEP was identified using a trypan blue stain (Figure S2). Both EEP and N-EEP can induce cell death in a short period (3 h) (Figure 2A). The long-term cytotoxic consequences of EEP and N-EEP were identified with the help of an MTT assay. The cell viability percentage of EEP and N-EEP was 24.6 ± 1.4% and 20.93 ± 0.14% at the highest treatment dose of 200 μg/mL, respectively. Both EEP and N-EEP have decreased the in vitro cell viability in a dose-dependent fashion (Figure 2B). Phytochemicals are nowadays formulated using liposomes as cargo to improve their bioavailability [48]. In a previous study, a hydro-ethanolic extract of ripe *Ceratonia siliqua* pods was prepared, which is capable of showing antioxidant properties due to hydroxybenzoic acid and flavonoid derivatives identified by LC-MS. The liposome-based topical formulation was explored to enhance the application of the extract in therapy. A representative skin cell line and erythrocytes were used to demonstrate their biocompatibility. The extract proved its antioxidant activity by using free radical scavenging, ferric ion reduction, and oxidative damage protection as test methods [49].

### 3.5. Hoechst Staining

**Figure 3:**
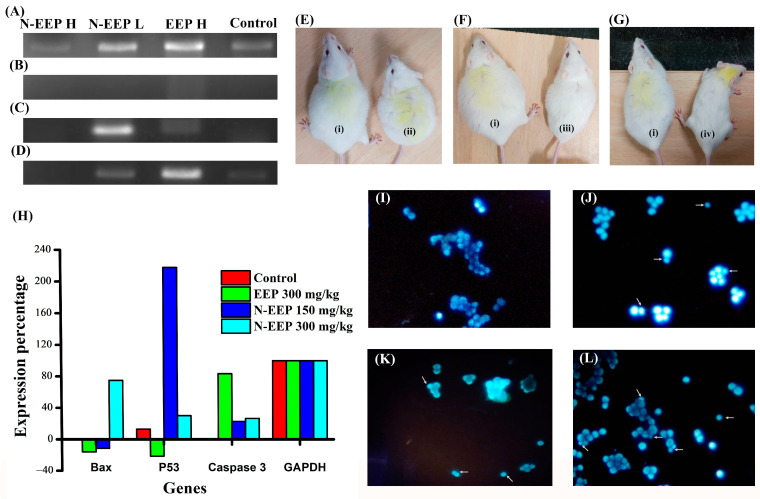
Gene expression was estimated in DLA cells aspirated from the mice tumor after treatment with EEP (high-300 mg/kg) and different concentrations of N-EEP (low and high, 150 and 300 mg/kg, respectively). The untreated tumor mice were taken as control. (**A**) GAPDH; (**B**) Bax; (**C**) p53; and (**D**) caspase 3. Representative images of animals after 29 days of treatment compared with the control. (**E**) (**i**) control, (**ii**) EEP 300 mg/kg; (**F**) (**i**) control, (**iii**) N-EEP 150 mg/kg; (**G**) (**i**) control, (**iv**) N-EEP 300 mg/kg. (**H**) The gene expression percentage of caspase 3, Bax, p53, and GAPDH in untreated control, EEP 300 mg/kg, N-EEP 150 mg/kg, and 300 mg/kg. Fluorescent microscopic images (20×) of Hoechst-stained cells in (**I**) control, (**J**) EEP 300 mg/kg treatment, (**K**) N-EEP 150 mg/kg treatment, and (**L**) N-EEP 300 mg/kg treatment. The arrows indicate the condensed nuclei indicating the apoptotic cells.

Condensed nuclei are typical indicators of apoptotic cells, which can be observed after staining and observing under a fluorescent microscope with a nuclear stain (Hoechst 33342) [50]. The Hoechst stain was used to identify the apoptosis by staining the nucleus. Figure 3I–L is a typical representative image for the Hoechst staining study, where we have withdrawn the cells in triplicate from the animals, stained them, and taken the typical snapshots at five random places for each slide. Table 4 shows the percentage of apoptotic cells after treatment with different concentrations of EEP and N-EEP counted under a fluorescent microscope post-Hoechst staining.

We could see that, after treatment with N-EEP and EEP at 300 μg/mL, many cells were present with condensed nuclei, indicating higher apoptosis induction in both EEP and N-EEP at high doses. Moderate apoptosis was induced after 150 μg/mL of N-EEP treatment. Our previous study found that the suppression of apoptosis in V79 cells chronically exposed to oxidative stress could lead to cisplatin resistance, as observed using Hoechst staining [35].

### 3.6. Effect of EEP and N-EEP on Tumor Growth

Dalton’s lymphoma ascitic (DLA) tumor is a liquid tumor that develops in the peritoneal cavity of mice. It is a cancerous tumor and grows rapidly inside the animal, killing it in nearly 15–20 days. The DLA cells can be aspirated from the peritoneal cavity of the induced animal when the tumor develops, diluted with sterile PBS, and injected into a new animal for tumor propagation. This model has been used for a few decades to study the effect of anticancer agents [33,37]. The effect of EEP and N-EEP on tumor growth was analyzed by measuring the body weight and volume of tumors in the mice. Every week, images of the untreated and treated mice were captured (Figure S3). It was observed that the tumor volume increased in all the groups as the days proceeded, but at different rates. The feed and water intake were monitored throughout the day before and 19 days after cancer induction, as mentioned in Supplementary Tables S1 and S2. There was a significant decrease in the feed intake of all the treatment groups except for N-EEP 150 mg/kg after 20 days of treatment. The percentages of decrease in feed intake were 48%, 15%, and 17% for the control, EEP, and N-EEP 300 mg/kg treatment groups, respectively, from day 1 to day 20. This indicates that the reduction in the percentage of feed intake was minimal in the EEP and N-EEP treatment compared to that of the control. No significant difference was observed for the same dose treatment (300 mg/kg) between the EEP and N-EEP groups. On the contrary, there was a 20% increase in the percentage of feed intake in N-EEP 150 mg/kg treated animals. The water intake was also decreased in the EEP 300 mg/kg treatment group and control group at the end of 20 days of treatment. However, there was a significant increase in water intake in the N-EEP 150 mg/kg and 300 mg/kg treatment groups, and the percentage of increase was 23.5% and 40%, respectively. This indicates the N-EEP 300 mg/kg positively affects feed and water intake after cancer induction and treatment. On the 20th day after tumor induction and treatment, the weight gain was found to be 22.1 ± 3.40 g for the untreated control. However, for the EEP 300 mg/kg, N-EEP 150 mg/kg, and N-EEP 300 mg/kg groups of animals, the weight gain was 14.74 ± 2.24 g, 11.7 ± 2.78 g, and 9.4 ± 1.36 g, respectively. There was a significant effect on the increase in body weight and tumor growth in the N-EEP 300 mg/kg treated group, in comparison with the control and EEP group at the same dose (300 mg/kg) (Figure 2E). Also, the tumor volume increment was slower in the N-EEP 300 mg/kg treated group compared to other groups (Figure 2C). On the 20th day, the volume of the tumor in the untreated control animals was 23.007 ± 1.15 cm^3^, whereas the tumor volume for EEP 300 mg/kg, N-EEP 150 mg/kg, and N-EEP 300 mg/kg was 19.38 ± 0.96 cm^3^, 18.77 ± 0.93 cm^3^, 16.57 ± 0.82 cm^3^, respectively. The average life span of all the groups was measured, and the life span of animals treated with N-EEP 300 mg/kg was significantly higher (Figure 2D). The percentage increase in life span compared to the control group is given in Table 5. The animals in the untreated control group survived up to 19.67 ± 1.63 days, whereas N-EEP 300 mg/kg treated animals survived for 27.25 ± 0.53 days, indicating a considerable leap in survival. The evidence of a 38.55% increase in life span observed for the N-EEP 300 mg/kg-treated animals was very promising to infer a drug candidate that is highly effective in controlling cancerous tumors in vivo.

Several Ayurvedic herbs have been studied for their antitumor properties and chemopreventive potential, including *Tinospora cordifolia (Wild) Miers ex Hook.f and Thomas*, *Ocimum sanctum* L., *Zizyphus mauritiana Lam.*, and *Curcuma longa* L., using a DLA mice model. The volume of the tumor, survival time, and hematological indices were assessed. The results indicated that crude herb oral administration enhanced survival and significantly reduced the volume of peritoneal ascitic fluid [51]. In another study, researchers used DLA cells to show the antitumor activity of *Luffa acutangular* extract. They found that the extract could inhibit tumor growth in a dose-dependent manner, similar to the standard drug cisplatin [52]. *Phyllanthus amarus* extract was explored to study the antitumor activity using DLA-induced mice, and it was found that the tumor cells were killed by apoptosis, evidenced by the gene expression of caspase 3 and bcl2 [53].

In a recent study, the effectiveness of *Pleurotus eous* aqueous extract (PEAE) in inhibiting tumor growth was studied in mice with DLA tumors. The results showed a dose-dependent reduction in tumor burden. At 500 and 1000 mg/mL concentrations, PEAE significantly decreased tumor volume by 62.47% and 72.13%, respectively. It also reduced tumor weight by 53.32% and 73.72% and improved longevity by 30.89% and 58.14% [39].

### 3.7. Analysis of Gene Expression

The expression of the major apoptosis-inducing gene was estimated in the DLA after 19 days of treatment in the tumor-bearing mice. Figure 3A–D gives the expression of GAPDH, Bax, p53, and caspase 3, respectively. Figure 3H represents the expression percentage of these genes. The study results show that the EEP 300 mg/kg treated group had a significant down-regulation of p53, whereas caspase 3 and Bax were upregulated compared to that of the control. This indicates that EEP treatment can promote apoptosis. On the other hand, the N-EEP 300 mg/kg treated group shows an elevation in the expression of Bax, p53, and caspase 3 compared to that of the control, indicating that N-EEP 300 mg/kg treatment can also promote apoptosis and suppress tumor growth. The expression of p53 was higher in the N-EEP 150 mg/kg treated group compared to all other treatment groups, but the Bax expression was lower compared to the N-EEP 300 mg/kg treatment group. The caspase 3 was also upregulated compared to that of the control in the N-EEP 150 mg/kg treated group. p53 plays a major part in the induction of apoptosis via various mechanisms apart from tumor suppression; it is also associated with the elicitation of Bax expression. The upregulation of Bax leads to an increase in mitochondrial outer membrane permeability, resulting in the release of Apaf1, caspase 9, and cytochrome c, which are the precursors of apoptosome formation. Apoptosome formation causes the activation of caspase 3, triggering the caspase-dependent apoptotic pathway. Upregulation of caspase 3 leads to the cleavage of DNA through the caspase-activated DNases.

Recent studies indicate that about one-third of cancer-related deaths are linked to diet, with balanced nutrition playing a crucial role in cancer prevention by reversing epigenetic abnormalities. A proper diet in cancer patients can influence gene expression and improve therapy effectiveness. Nutraceuticals act as potent antioxidants and anticancer agents at the cellular level. Natural compounds like resveratrol, curcumin, sulforaphane, quercetin, indole-3-carbinol, epigallocatechin-3-gallate (EGCG), astaxanthin, and lycopene influence epigenetic modifications, including histone modification via HDAC and HAT inhibition, DNMT inhibition, and non-coding RNA expression. These compounds help regulate the epigenome, reducing cancer risk [54]. Combining plant extracts with chemotherapy enhances anticancer effects by targeting pathways such as PI3K/AKT, NF-κB, JNK, ERK, and WNT/β-catenin [55].

Specific natural compounds demonstrate notable anticancer properties. EGCG from tea modulates miR-16 and miR-21 to inhibit proliferation. DIM from cruciferous vegetables downregulates miR-92a, affecting NF-κB and preventing cancer progression. Glyceollins regulate miR-181d, miR-181c, and tumor-suppressing miRNAs like miR-22, 29b/c, 34a, 30d, and 195. Quercetin induces apoptosis by regulating miR-16, 34a, 26b, let-7g, 125a, and miR-605 while reducing metastasis-associated miRNAs like miR-146a/b, 503, and 194 [56]. In our future studies, we shall further explore the in-depth mechanism of tumor reduction and enhanced survival.

In summary, EEP and N-EEP treatment induces apoptosis through the mitochondrial-mediated intrinsic pathway [57]. The results are supported by the expression percentage graph (Figure 3H) and the agarose gel electrophoresis image (Figure 3A–D). Thus, our nanoformulation (N-EEP) at a low dose (150 mg/kg) can elicit tumor suppression at a higher rate, whereas at a high dose (300 mg/kg), tumor suppression and activation of the caspase-dependent apoptotic pathway are both triggered compared to the control mice. In this manner, a synergistic effect of tumor suppression and apoptosis activation resulted in a massive decrease in tumor volume of the N-EEP 300 mg/kg treatment mice.

## 4. Conclusions

In the present study, we have demonstrated that both EEP and N-EEP inhibit cancer cell migration in A375 cells and reduce cell viability in DLA cells, with a more pronounced effect observed for N-EEP at the same dosage. N-EEP was shown to be highly biocompatible compared to EEP, as evidenced by a hemocompatibility assay. Furthermore, N-EEP significantly reduced tumor cell growth and prolonged the lifespan of DLA-bearing mice compared to EEP and control groups. It has been established that N-EEP follows the mitochondrial-mediated, caspase-dependent apoptotic pathway and can arrest the cell cycle by increasing p53 gene expression. After nanoformulation, EEP showed a significant improvement in its anticancer activity against DLA. Our findings reveal the considerable potential of N-EEP as an anticancer agent in both in vitro and in vivo models. Further research is necessary to fully understand the complex mechanisms of action and identify the specific bioactive constituents present in N-EEP.

## Figures and Tables

**Table 1:** Experimental groups, treatment, and dosage.

Experimental Group	Treatment/Dose	No. of Mice and Sex
Group 1 (n = 6)	Control	N = 6 (Female)
Group 2 (n = 6)	EEP 300 mg/kg	N = 6 (Female)
Group 3 (n = 6)	N-EEP 150 mg/kg	N = 6 (Female)
Group 4 (n = 6)	N-EEP 300 mg/kg	N = 6 (Female)

**Table 2:** Primer sequence and amplification of the gene from cDNA.

Gene	Primer Sequence	PCR Amplification (49 Cycles)		
		Denaturation	Annealing	Extension
GAPDH	5′-TGCCTCCTGCACCACCAA-3′ (forward) 5′-GCCTGCTTCACCACCTTC-3′ (reverse)	95 °C for 30 s	49 °C for 30 s	72 °C for 1 min
Bax	5′-GTTTCATCCAGGATCGAGCAG-3′ (forward) 5′-CATCTTCTTCCAGATGGT-3′ (reverse)	95 °C for 30 s	49 °C for 30 s	72 °C for 1 min
p53	5′-CGGAGGTCGAGACGCTG-3′ (forward) 5′-CACATGTACTTGTAGTGGATGGTGG-3′ (reverse)	95 °C for 30 s	57 °C for 30 s	72 °C for 1 min
Caspase-3	5′-GGAAACCAACAGTAGTCAGTCCT-3′ (forward) 5′-GCGAGTGAGAATGTGCATAAATTC-3′ (Reverse)	95 °C for 30 s	49 °C for 30 s	72 °C for 1 min

**Table 3:** Percentage of area closure.

Groups	V79	A375
Control	100%	97.1 ± 0.9%
EEP 100 μg/mL	99.4 ± 0.23%	44.6 ± 8.58%
N-EEP 100 μg/mL	100%	13.5 ± 5.9%

**Table 4:** The percentage of apoptotic cells after treatment with different concentrations of EEP and N-EEP assessed using Hoechst staining.

Sample	Percentage of Apoptotic Cells ± S.E.
Untreated control group	11 ± 2
EEP (300 mg/kg) group	36 ± 4
N-EEP (150 mg/kg) group	52 ± 4
N-EEP (300 mg/kg) group	69 ± 5

**Table 5:** The life span increase was documented for animals treated with EEP and N-EEP compared to the untreated control group.

Treatment Groups	% Life Span Increase
EEP (300 mg/kg)	8.05 ± 3.48
N-EEP (150 mg/kg)	19.49 ± 2.18
N-EEP (300 mg/kg)	38.55 ± 5.69

## Data Availability

The data presented in this study are available on request from the corresponding author due to privacy.

## Supplementary Materials

The following supporting information can be downloaded at: https://www.mdpi.com/article/10.3390/pharmaceutics17020270/s1, Figure S1. SEM image of (A) EEP and (B) N-EEP (C) the stability of the liposomes at different temperatures; Figure S2. Trypan blue staining of DLA cells after treated with different concentration of EEP and N-EEP; Figure S3. Images of mice from Day1 to Day 19 after cancer induction and during the treatment with EEP and N-EEP; Table S1: Average feed intake of each animal in grams over 20 days following cancer induction; Table S2: Average water intake for each animal presented in milliliters over 20 days following cancer induction.
