# Supplementary material for: Inhibitory Effect of Nano-Formulated Extract of Passiflora incarnata on Dalton’s Lymphoma Ascites-Bearing Swiss albino Mice
Source: Pharmaceutics. 2025 Feb 18;17(2):270. doi: 10.3390/pharmaceutics17020270 (PMC11859039; doi:10.3390/pharmaceutics17020270)
Supplement: Supplementary file 1 [file pharmaceutics-17-00270-s001.zip › pharmaceutics-3433504-supplementary.pdf]

Supplementary Information

Inhibitory Effect of Nano-formulated Extract of *Passiflora incarnata* on Dalton's Lymphoma Ascites lymphoma-bearing *Swiss albino* Mice

Balasubramanian Deepika, Janani Gopalarethinam, Devadass Jessy Mercy, Saranya Udayakumar, Agnishwar Girigoswami, Koyeli Girigoswami*

S1(A)

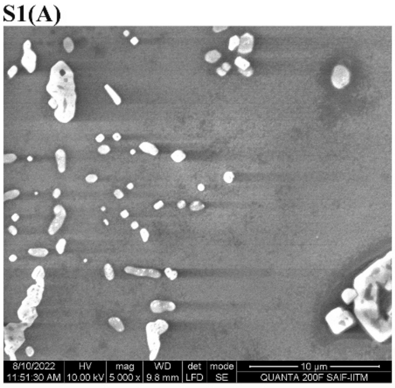

EEP

S1(B)

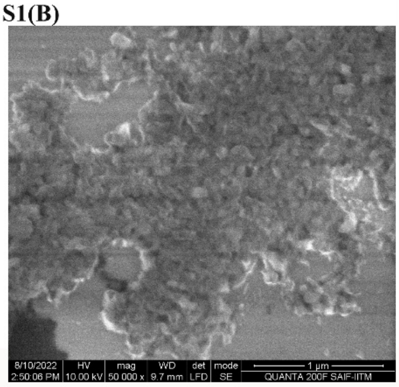

N-EEP

S1(C)

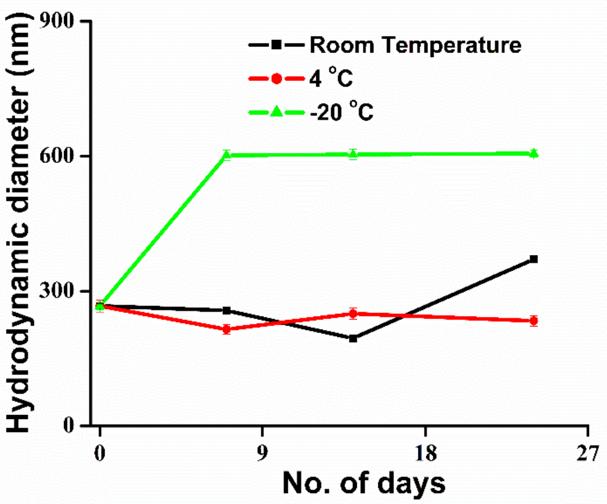

Figure S1. SEM image of (A) EEP and (B) N-EEP (C) the stability of the liposomes at different temperatures

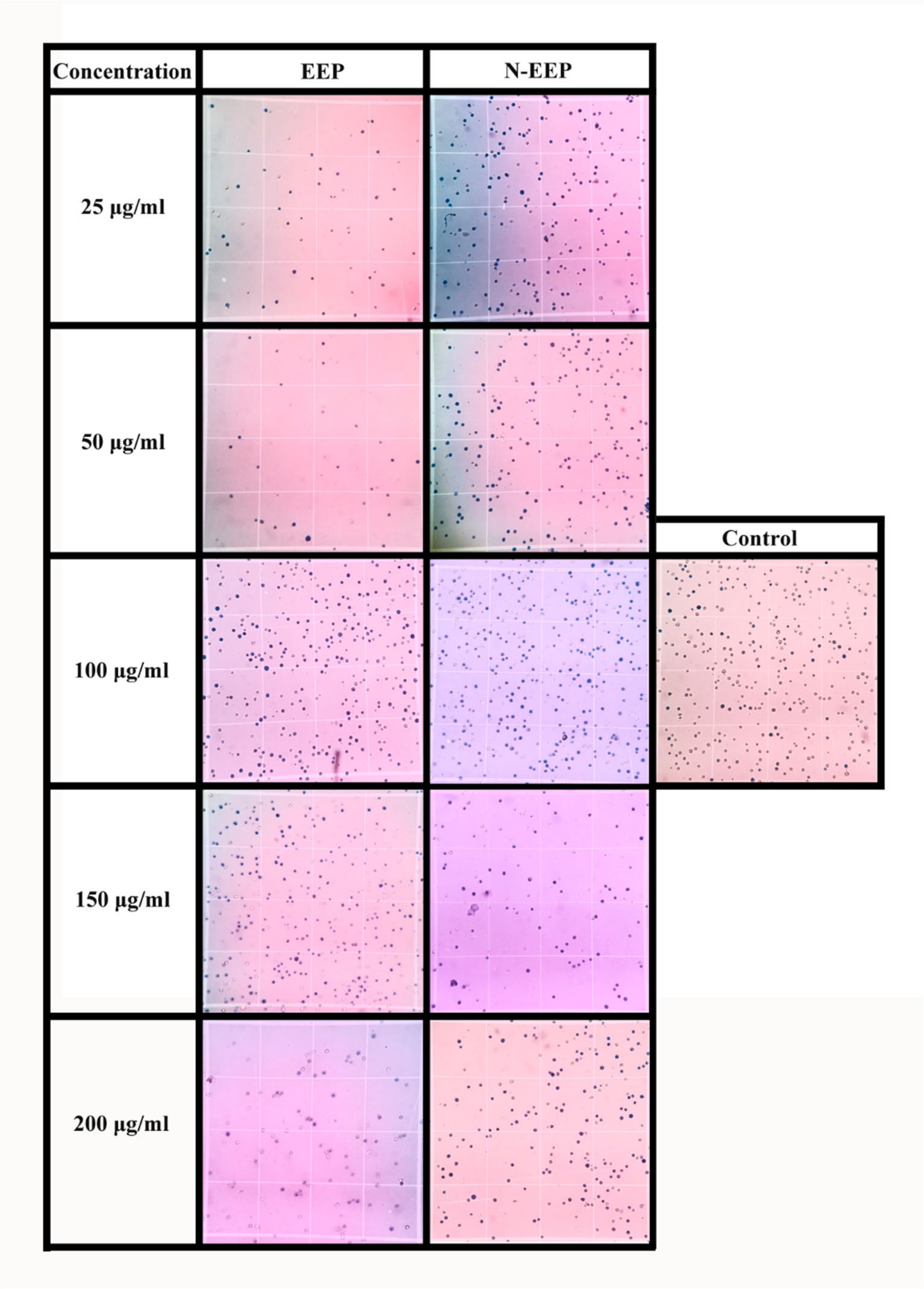

Figure S2. Trypan blue staining of DLA cells after treated with different concentration of EEP and N-EEP

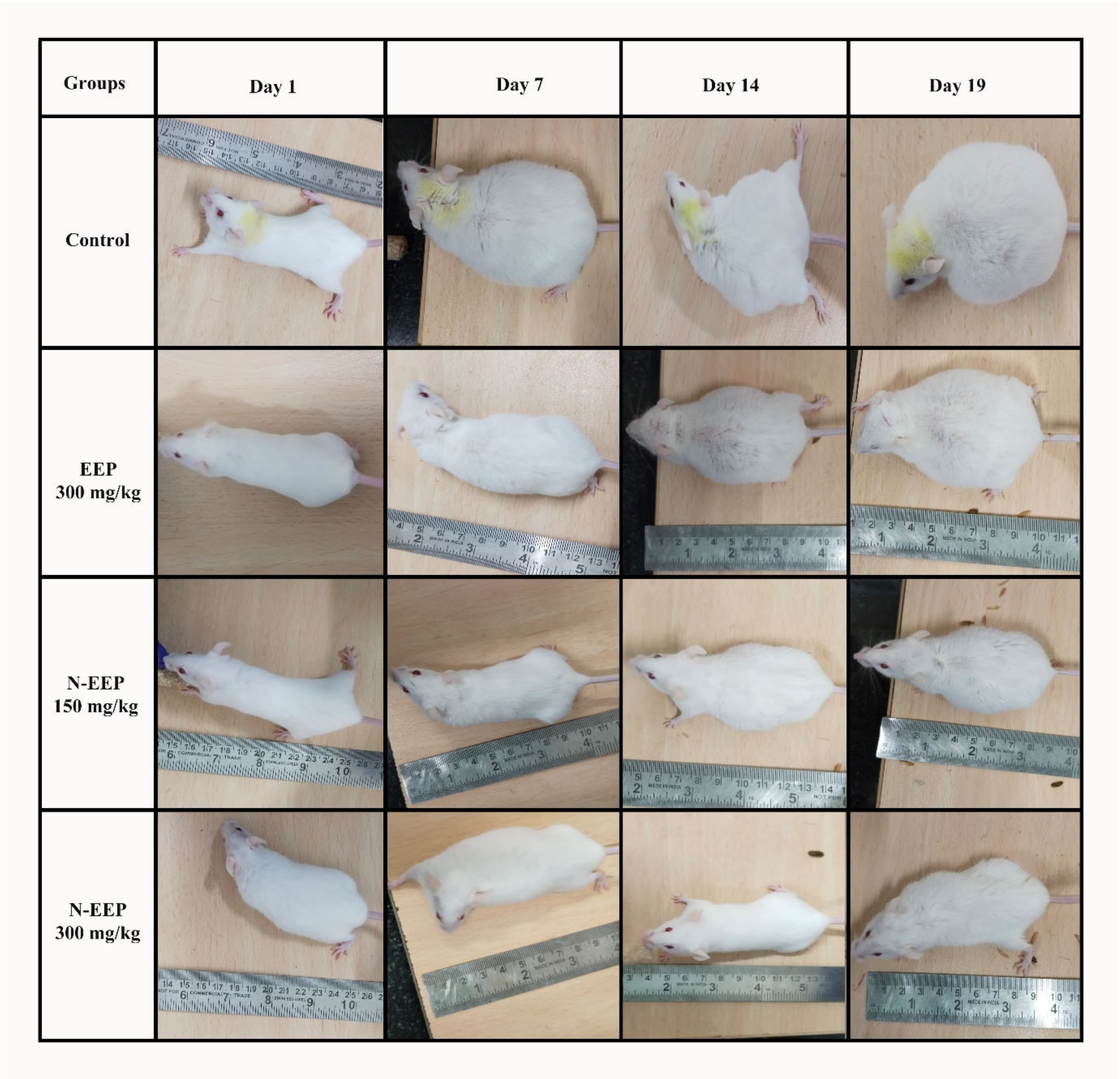

Figure S3. Images of mice from Day1 to Day 19 after cancer induction and during the treatment with EEP and N-EEP

Table S1: Average feed intake of each animal in grams over 20 days following cancer induction.

Days	Control (g)	EEP-300 mg/kg (g)	N-EEP-150 mg/kg (g)	N-EEP-300 mg/kg (g)
Day 1	5 ± 0.04	4.567 ± 0.68	3.867 ± 0.09	4.267 ± 0.18
Day 2	4.667 ± 0.09	4.034 ± 0.16	4.234 ± 0.30	3.867 ± 0.18
Day 3	4.934 ± 0.14	4.167 ± 0.35	4.1 ± 0.02	4.5 ± 0.25
Day 4	4.4 ± 0.37	3.5 ± 0.73	3.6 ± 0.04	4.034 ± 0.36
Day 5	6.2 ± 1.93	6.534 ± 2.07	5.3 ± 1.62	5.284 ± 1.61
Day 6	3.767 ± 0.11	3.334 ± 1.13	3.267 ± 0.42	2.917 ± 0.27
Day 7	3.2 ± 0.28	2.634 ± 0.73	2.5 ± 0.54	3.467 ± 0.47
Day 8	3.434 ± 0.16	2.9 ± 0.58	2.934 ± 0.23	3.2 ± 0.04
Day 9	4.217 ± 0.86	2.534 ± 0.94	2.467 ± 0.18	3.367 ± 0.54
Day 10	3.717 ± 0.22	3.134 ±0.51	3.434 ± 1.06	4.1 ± 0.68
Day 11	2.5 ± 0.44	4.067 ± 0.28	3.5 ± 0.82	3.5 ± 0.11
Day 12	4.067 ± 0.47	3.534 ± 0.23	4.034 ± 0.02	3.534 ± 0.56
Day 13	3.017 ± 0.10	4.117 ± 1.11	3.184 ± 0.29	2.6 ± 0.7
Day 14	3.584 ± 0.01	4.917 ± 0.88	4.15 ± 0.5	3.634 ± 0.02
Day 15	3.4 ± 0.23	2.94 ± 1.5	3.767 ± 0.07	3.534 ± 0.32
Day 16	2.667 ± 0.14	3.12 ± 0.67	4.44 ± 0.02	3.267 ± 0.14
Day 17	2.667	3.36 ± 0.6	4.56 ± 0.34	4.36 ± 0.53
Day 18	2.4 ± 0.28	2.7	5.04 ± 1.7	5.05 ± 0.82
Day 19	1.9 ± 0.21	3.35 ± 0.89	1.9 ± 0.28	2.25 ± 1.23
Day 20	2.6 ± 1.83	3.867 ± 1.23	4.65 ± 0.53	3.5 ± 1.64

Table S2: Average water intake for each animal presented in milliliters over 20 days following cancer induction.

Days	Control (ml)	EEP-300 mg/kg (ml)	N-EEP-150 mg/kg (ml)	N-EEP-300 mg/kg (ml)
Day 1	2.83 ± 0.12	5 ± 0.047	2.67	2.5 ± 0.12
Day 2	2.83 ± 0.59	3.67 ± 0.70	2.67 ± 0.24	2
Day 3	2.17 ± 0.12	4.67 ± 0.47	1.83 ± 0.12	3 ± 0.70
Day 4	4.83 ± 1.30	4.67 ± 0.70	3.5 ± 0.12	2 ± 0.47
Day 5	3.58 ± 2.30	5.83 ± 1.53	3.67 ± 1.18	3.5 ± 0.82
Day 6	2.17 ± 0.12	4.83 ± 0.35	2.5 ± 0.59	4 ± 0.47
Day 7	3.17 ± 0.12	3.67 ± 0.47	1.5 ± 1.06	2.84 ± 0.35
Day 8	2.33 ± 0.24	2.5 ± 0.58	2.84 ± 0.12	3.83 ± 1.06
Day 9	1.5 ± 0.58	2.5 ± 0.12	1.67 ± 0.35	1.83 ± 0.12
Day 10	2.5 ± 0.35	3.33 ± 0.70	4 ± 1.41	3.16 ± 0.35
Day 11	1.67	5.67 ± 1.17	2.33 ± 1.18	2.67 ± 0.24
Day 12	2.67 ± 0.47	3.83 ± 0.35	1.67 ± 1.17	2.67 ± 1.17
Day 13	2.67 ± 0.23	3.33 ± 0.23	1.83 ± 0.82	2.5 ± 1.06
Day 14	2.67 ±0.24	2.33 ± 0.47	2.5 ± 0.82	2.17 ± 0.82
Day 15	4.17 ± 1.06	2.5 ± 0.35	1.5 ± 0.35	2.17 ± 0.59
Day 16	2.83 ± 0.12	1.83 ± 0.12	1 ± 0.70	2.5 ± 0.59
Day 17	2.5 ± 0.12	1.83 ± 0.35	2 ± 0.24	1.67 ± 0.70
Day 18	2.75 ± 0.18	4.25 ± 0.88	3 ± 0.70	3.25 ± 0.53
Day 19	2.75 ± 0.18	1.75 ± 0.53	1.5 ± 0.35	1.75 ± 0.53
Day 20	2 ± 1.41	1.5 ± 1.06	3.3 ± 1.62	3.5 ± 2.4
